# CG-NAP/Kinase Interactions Fine-Tune T Cell Functions

**DOI:** 10.3389/fimmu.2019.02642

**Published:** 2019-11-12

**Authors:** Navin Kumar Verma, Madhavi Latha Somaraju Chalasani, John D. Scott, Dermot Kelleher

**Affiliations:** ^1^Lee Kong Chian School of Medicine, Nanyang Technological University Singapore, Singapore, Singapore; ^2^Autoimmunity and Inflammation Program, Hospital for Special Surgery, New York, NY, United States; ^3^Department of Pharmacology, University of Washington School of Medicine, Seattle, WA, United States; ^4^Departments of Medicine and Biochemistry and Molecular Biology, University of British Columbia, Vancouver, BC, Canada

**Keywords:** adaptor protein, kinases, CG-NAP, AKAP450, T cell motility, immune synapse

## Abstract

CG-NAP, also known as AKAP450, is an anchoring/adaptor protein that streamlines signal transduction in various cell types by localizing signaling proteins and enzymes with their substrates. Great efforts are being devoted to elucidating functional roles of this protein and associated macromolecular signaling complex. Increasing understanding of pathways involved in regulating T lymphocytes suggests that CG-NAP can facilitate dynamic interactions between kinases and their substrates and thus fine-tune T cell motility and effector functions. As a result, new binding partners of CG-NAP are continually being uncovered. Here, we review recent advances in CG-NAP research, focusing on its interactions with kinases in T cells with an emphasis on the possible role of this anchoring protein as a target for therapeutic intervention in immune-mediated diseases.

## Introduction

T lymphocytes play a central role in immune defense by mounting specific responses to eliminate infections and transformed cells. To perform an immunosurveillance function, T cells continuously circulate throughout the body until they encounter specific antigen on the surface of the antigen presenting cell (APC, Table 1). Such contact with an APC triggers an initial activation of the T cell, which rapidly reorients its organelles and mobilizes signaling proteins to the contact site. This process is accompanied by dynamic structural and cytoskeletal changes within the T cell. An activated T cell undergoes an episode of rapid proliferation, cytokine secretion, differentiation into effector subtypes, and site-specific recruitment. These functional processes in T lymphocytes are precisely regulated and are critical for mounting an effective immune response.

**Table 1:** A list of abbreviations used.

**Abbreviations used**	
AKAP	A-kinase anchoring protein
APC	Antigen presenting cell
cAMP	Cyclic adenosine monophosphate
CAMSAP2	Calmodulin regulated spectrin associated protein family member 2
CAMSAP3	Calmodulin regulated spectrin associated protein family member 3
CBFA2T3	CBFA2/RUNX1 partner transcriptional co-repressor 3
Cdk	Cyclin dependent kinase
CG-NAP	Centrosome- and Golgi-localized protein kinase N-associated protein
CHO	Chinese hamster ovary
CK1δ/ε	Casein kinase 1 delta/epsilon
CRISPR	Clustered regularly interspaced short palindromic repeats
EB1	End-binding protein 1
GM130	130 KDa cis-Golgi matrix protein
GTP	Guanosine triphosphate
HIV	Human immunodeficiency viruses
ICAM-1	Intercellular adhesion molecule 1
IL	Interleukin
IS	Immune synapse
JC virus	John Cunningham virus
KCNE1	Potassium voltage-gated channel subfamily E regulatory subunit 1
Kiz	Kizuna
LAT	Linker for activation of T cells
LFA-1	Lymphocyte function-associated antigen 1
MAPRE1	Microtubule associated protein RP/EB family member 1
MMG8	Myomegalin variant 8
MTCL1	Microtubule cross-linking factor 1
MTOC	Microtubule organizing center
NFAT	Nuclear factor of activated T cells
NFκB	Nuclear factor kappa B
PACT	Pericentrin-AKAP450 centrosomal targeting
PDE4D	Phosphodiesterase 4D
PKA	Protein kinase A
PKC	Protein kinase C
PKN	Protein kinase N
PLCγ1	Phospholipase C gamma 1
Plk1	Polo-like kinase 1
PP1	Protein phosphatase 1
PP2A	Protein phosphatase 2A
PR130	Serine/Threonine-protein phosphatase 2a 72/130 KDa regulatory subunit B
RAPGEF2	Rap guanine nucleotide exchange factor 2
RUNX1T1	RUNX1 partner transcriptional co-repressor 1
siRNAs	Small interfering RNA
TCR	T cell receptor
TGFβ	Transforming growth factor beta
TUBGCP	Tubulin gamma complex associated protein
γ-TuRC	Gamma tubulin ring complex

Multiple stages of T cell functions, such as activation, differentiation, conjugate formation with APCs, homing and motility are crucially regulated by protein kinases (1, 2). As members of the kinase superfamily are widely distributed within cells and often have broad substrate specificity, a crucial element in signal transduction is local control of substrate specificity (3, 4). How is an individual kinase directed to connect with a single substrate or multiple components of a pool of downstream substrates? In some cases, kinase specificity is achieved by influencing substrate recognition. In this context, a class of proteins collectively known as “adaptor, anchoring and scaffolding proteins,” have emerged as important mediators of signal transduction processes (5, 6). These proteins form specialized docking platforms that facilitate the formation of multicomponent signaling complexes, maintain static protein-protein interactions, position their kinase cargo in proximity to a subset of substrates, organize processes and components of protein kinase cascades and thus streamline cell signaling responses (6–13). In T lymphocytes, these signal-organizing proteins allow signals to be transduced with precision in response to molecular instructions from the cell surface (14–16). Most importantly, these anchoring/adaptor proteins facilitate the phosphorylation and dephosphorylation of protein kinases, including trans- and auto-phosphorylations, which are important for the kinases to gain catalytically competent conformation in order to respond to intra- and/or extra-cellular signals (16, 17). Anchoring/adaptor proteins thus control numerous cellular processes in T lymphocytes, including cell fate decisions, activation, differentiation and various stages of development and functions (16–19). Herein, we review the involvement of such an anchoring protein “Centrosome and Golgi localized protein kinase N (PKN)-Associated Protein” (CG-NAP), also known as A-Kinase Anchoring Protein 450 (AKAP450) (20–22), in the regulation of protein kinase dynamics and functional outcomes in T cells.

## A-Kinase Anchoring Proteins (AKAPs)

AKAPs are a family of ubiquitously expressed structurally diverse signal-organizing proteins with tissue/cell-type specific expression patterns in human. So far, 41 AKAPs encoded by 41 genes have been experimentally validated in human cells and tissues (8, 23) (Table 2). Nine different AKAPs have been described in human T lymphocytes – AKAP1, AKAP2, AKAP5, AKAP8, AKAP9 (known as CG-NAP), AKAP11, Ezrin, RUNX1T1, and RUNX1T3 (24–26) and at least eight AKAPs with apparent molecular masses of 60, 75, 95, 120, 165, 190, 245, and 275 kDa were detected in mouse T lymphocytes (27); however, their involvements in the regulation of immune functions remain poorly understood.

**Table 2:** A list of AKAP family proteins.

**S.N**.	**AKAPs**	**Name**	**Synonyms**	**HGNC ID (gene)**	**Chromosome**
1.	AKAP1	A-kinase anchoring protein 1	PRKA1, AKAP121, AKAP149, SAKAP84, S-AKAP84, AKAP84, D-AKAP1, PPP1R43, TDRD17	HGNC:367	17q22
2.	AKAP2	A-kinase anchoring protein 2	PRKA2, AKAP-KL, KIAA0920, DKFZp564L0716, MISP2	HGNC:372	9q31.3
3.	AKAP3	A-kinase anchoring protein 3	FSP95, SOB1, AKAP110, CT82	HGNC:373	12p13.32
4.	AKAP4	A-kinase anchoring protein 4	p82, hAKAP82, AKAP82, Fsc1, HI, CT99	HGNC:374	Xp11.22
5.	AKAP5	A-kinase anchoring protein 5	AKAP75, AKAP79	HGNC:375	14q23.3
6.	AKAP6	A-kinase anchoring protein 6	KIAA0311, mAKAP, AKAP100, PRKA6, ADAP6	HGNC:376	14q12
7.	AKAP7	A-kinase anchoring protein 7	AKAP18, AKAP15	HGNC:377	6q23.2
8.	AKAP8	A-kinase anchoring protein 8	AKAP95, DKFZp586B1222	HGNC:378	19p13.12
9.	AKAP9	A-kinase anchoring protein 9	KIAA0803, AKAP350, AKAP450, CG-NAP, YOTIAO, HYPERION, PRKA9, MU-RMS-40.16A, PPP1R45, LQT11	HGNC:379	7q21.2
10.	AKAP10	A-kinase anchoring protein 10	D-AKAP2, PRKA10, MGC9414	HGNC:368	17p11.2
11.	AKAP11	A-kinase anchoring protein 11	KIAA0629, AKAP220, PRKA11, FLJ11304, DKFZp781I12161, PPP1R44	HGNC:369	13q14.11
12.	AKAP12	A-kinase anchoring protein 12	AKAP250, SSeCKS, gravin	HGNC:370	6q25.1
13.	AKAP13	A-kinase anchoring protein 13	LBC, Ht31, BRX, AKAP-Lbc, c-lbc, PROTO-LB, HA-3, ARHGEF13	HGNC:371	15q25.3
14.	AKAP14	A-kinase anchoring protein 14	AKAP28	HGNC:24061	Xq24
15.	AKAP17A	A-kinase anchoring protein 17A	CXYorf3, SFRS17A, XE7, XE7Y, DXYS155E, MGC39904, 721P, CCDC133	HGNC:18783	Xp22.33 and Yp11.32
16.	AKAP17BP	A-kinase anchoring protein 17B, pseudogene	AKAP16B, AKAP16BP	HGNC:38514	Xq24
17.	ACBD3	Acyl-CoA binding domain containing 3	GOLPH1, GOCAP1, GCP60, PAP7	HGNC:15453	1q42.12
18.	ARFGEF2	ADP ribosylation factor guanine nucleotide exchange factor 2	BIG2	HGNC:15853	20q13.13
19.	CHD8	Chromodomain Helicase DNA Binding Protein 8	HELSNF1, Helicase with SNF2 Domain 1, AUTS18, Duplin, KIAA1564	HGNC:20153	14q11.2
20.	CMYA5	Cardiomyopathy associated 5	C5orf10, SPRYD2, DKFZp451G223, TRIM76	HGNC:14305	5q14.1
21.	C2orf88	Chromosome 2 open reading frame 88	MGC13057, smAKAP	HGNC:28191	2q32.2
22.	EZR	Ezrin	VIL2, Villin 2, P81, Cytovillin	HGNC:12691	6q25.3
23.	GSKIP	GSK3B Interacting Protein	C14orf129	HGNC:20343	14q32.2
24.	ITGA4	α4 integrin	CD49D	HGNC: 6140	2q31.3
25.	MAP2	Microtubule associated protein 2	MAP2A, MAP2B, MAP2C	HGNC:6839	2q34
26.	MSN	Moesin	IMD50, HEL70, Membrane-Organizing Extension Spike Protein	HGNC: 7373	Xq12
27.	MYO7A	Myosin VIIA	USH1B, DFNB2, DFNA11, SRD2	HGNC:7606	11q13.5
28.	MYRIP	Myosin VIIA and Rab interacting protein	DKFZp586F1018, exophilin-8, MyRIP, SLAC2-C, SLAC2C	HGNC:19156	3p22.1
29.	NBEA	Neurobeachin	KIAA1544, BCL8B, FLJ10197, LYST2	HGNC:7648	13q13.3
30.	NF2	Neurofibromin 2	merlin, ACN, SCH, BANF	HGNC:7773	22q12.2
31.	OPA1	Optic Atrophy Protein 1	MyRIP, Optic Atrophy Protein 1, OPA1 Mitochondrial Dynamin Like GTPase, NTG, NPG, BERHS, LargeG, MTDPS14, KIAA0567, Dynamin-Like Guanosine Triphosphatase	HGNC: 8140	3q29
32.	PDE4DIP	Phosphodiesterase 4D Interacting Protein	MMGL, CMYA2, Myomegalin, Cardiomyopathy-Associated Protein	HGNC:15580	1q21.2
33.	PIK3CG	Phosphatidylinositol-4,5-Bisphosphate 3-Kinase Catalytic Subunit Gamma	P110γ, PI3Kγ, PI3CG, P120-PI3K	HGNC:8978	7q22.3
34.	RAB32	Ras-Related Protein Rab-32	Rab32	HGNC:9772	6q24.3
35.	RSPH3	Radial Spoke Head 3	RSP3, RSHL2	HGNC:21054	6q25.3
36.	RUNX1T1	RUNX1 Partner Transcriptional Co-Repressor 1	AML1T1, CBFA2T1, MTG8	HGNC:1535	8q21.3
37.	RUNX1T3	RUNX1 Partner Transcriptional Co-Repressor 3	CBFA2T3, ETO2, HMTG16, MTG8-Related Protein 2, MTG16, MTGR2, ZMYND4	HGNC:1537	16q24.3
38.	SPHKAP	SPHK1 interactor, AKAP domain containing	SKIP	HGNC:30619	2q36.3
39.	SYNM	Synemin	DMN, KIAA0353, SYN	HGNC:24466	15q26.3
40.	TNNT2	Troponin T2	c-troponinT, TnTC, CMPD2, LVNC6, CMD1D, CMH2	HGNC:11949	1q32.1
41.	WASF1	WAS protein family member 1	WAVE1, SCAR1, KIAA0269, WAVE	HGNC:12732	6q21

Although members of the AKAP family differ greatly in their amino-acid sequences, structures, intracellular localizations and repertoire of protein binding partners, they all interact directly with the regulatory subunits of the protein kinase A (PKA) (28–33). However, the mechanism by which molecular interactions between specific AKAPs and PKA regulate normal and pathological signaling in human cells/tissues is just beginning to be understood.

AKAPs, through association with PKA, are involved in regulating T cell functions through the ubiquitous second messenger molecule cAMP (34–36), which controls cellular processes dictated by cell surface receptor-induced signaling (37–39). The interactions between AKAPs and PKA are complex as there are four distinct regulatory subunit isoforms of PKA – RIα, RIβ, RIIα, and RIIβ (7). These subunits differ in tissue distribution, cAMP sensitivity and AKAP-mediated localization, which fine-tune molecular signals depending on when and where PKA activity is applied (40). Most AKAPs bind to the RII isoform and a few dual-specific AKAPs can also interact with the RI isoform (33, 41). In addition, there are recent examples of RI selective AKAPs (42–44). Furthermore, most cell types simultaneously express multiple AKAPs (e.g., human T cells express at least 9 different AKAPs) (24–26).

It should be noted that the PKA-binding module of AKAPs denotes only one facet of their regulatory control. Apart from their interactions with PKA, AKAPs also interact with other downstream proteins and signaling enzymes, including protein kinase C (PKC) isoforms and PKN, protein phosphatases, phosphodiesterases, small GTPases (8) and substrates to integrate a diverse range of signals within distinct multivalent assemblies. The spatiotemporal interactions between enzymes and target substrates are important in the regulation of T cell functions as well as in the maintenance of T cell homeostasis (27).

## CG-NAP: A Giant Member of the AKAP Protein Family

**Figure 1:**
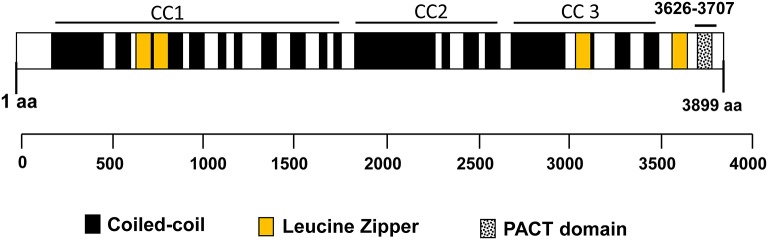
An illustration of the domain organization of the CG-NAP. Modular structures of coiled-coil (CC1, CC2, and CC3), 4 Leucine Zippers and the PACT domain are shown schematically.

CG-NAP is a member of the AKAP family, prominently expressed in human T cells, in which this giant protein predominantly localizes to the centrosome (20). The human *CG-NAP* gene is located on the chromosome 7q21-22 and contains at least 50 exons (45–47). A total of 16 splice variants have been identified in the *CG-NAP* gene (Table 3). The cDNA derived from the *CG-NAP* gene contains 11.7 kb open reading frame coding the 3899 amino acid protein with a calculated molecular mass of 451.8 kDa (45). The CG-NAP protein has several stretches of coiled-coil structures and four leucine zipper-like motifs (Figure 1) and these structural motifs are involved in interactions with other signaling proteins (e.g., PKA, PKN and PKC isoforms) (45). Amino acid sequence comparison using BLAST analysis shows that regions of human CG-NAP share high homology with the rabbit AKAP120 and limited homology to the mouse pericentrin (48–50).

**Table 3:** A list of 16 splice variants (transcripts) of the CG-NAP gene in human.

**S.N**.	**Name**	**Transcript ID**	**bp**	**Protein**	**Biotype**	**UniProt**
1.	AKAP9-201	ENST00000356239.7	12471	3907aa	Protein coding	Q99996
2.	AKAP9-203	ENST00000359028.6	12247	3910aa	Protein coding	A0A0A0MRF6
3.	AKAP9-202	ENST00000358100.6	10309	3126aa	Protein coding	A0A0A0MRE9
4.	AKAP9-216	ENST00000619023.4	6006	1637aa	Protein coding	A0A087WX84
5.	AKAP9-204	ENST00000394534.6	5652	1769aa	Protein coding	H7BYL6
6.	AKAP9-206	ENST00000435423.1	1904	318aa	Protein coding	H0Y6Q0
7.	AKAP9-213	ENST00000491695.1	1389	282aa	Protein coding	A0A2R8Y590
8.	AKAP9-205	ENST00000394564.5	1356	314aa	Protein coding	Q6PJH3
9.	AKAP9-207	ENST00000438114.1	696	232aa	Protein coding	H7BZV6
10.	AKAP9-214	ENST00000493453.1	6020	No protein	Retained intron	–
11.	AKAP9-211	ENST00000487258.5	3983	No protein	Retained intron	–
12.	AKAP9-208	ENST00000463118.1	891	No protein	Retained intron	–
13.	AKAP9-212	ENST00000487692.1	869	No protein	Retained intron	–
14.	AKAP9-215	ENST00000493976.1	724	No protein	Retained intron	–
15.	AKAP9-209	ENST00000484815.1	671	No protein	Retained intron	–
16.	AKAP9-210	ENST00000486313.1	584	No protein	lncRNA	–

*The list was prepared from the Ensembl database using the HGNC ID HGNC:379*.

## CG-NAP/Protein Kinase Interactions in T Cells

**Figure 2:**
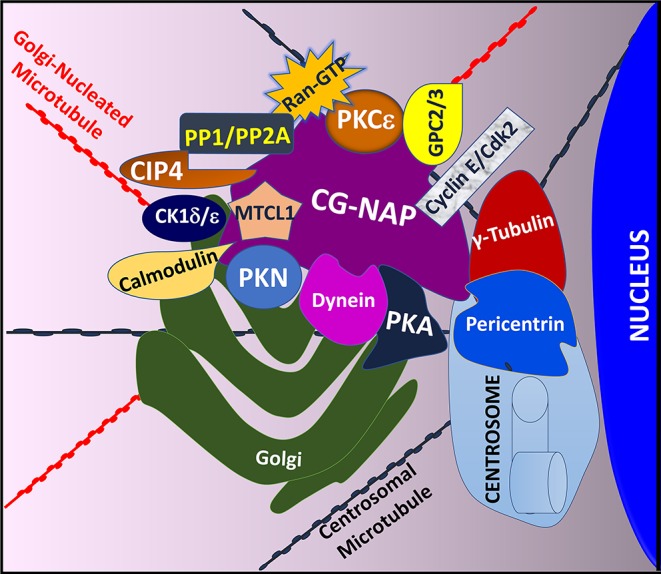
An illustration showing T cell CG-NAP as an anchoring protein that provides a docking platform for several kinases and their substrates. In particular, the Golgi-localized CG-NAP interacts with PKA, PKN, PKC isoforms, dynein, pericentrin, γ-tubulin, MTCL1, PP1, PP2A, CK1, calmodulin, CIP4, cyclin E, and Cdk2 and regulates T cell functions, including activation, proliferation, IS formation and migration.

The modular architecture of CG-NAP brings many protein kinases and their substrates in proximity within a cell and thus regulates the rate and magnitude of cytoplasmic catalysis. Since its initial characterization in 1999 (45) and the establishment of its role in regulating intracellular membrane trafficking and cell cycle progression (51), several interacting partners of CG-NAP have been identified in various cell types, including T lymphocytes (Figure 2).

Previous studies using co-immunoprecipitation approaches and deletion mutants to identify CG-NAP interacting partners revealed that CG-NAP functions as an anchoring molecular platform for protein kinases, including PKA (45). Using a yeast two-hybrid screening system, it has been demonstrated that CG-NAP interacts with the N-terminus of PKN (45). In addition, CG-NAP associates with the catalytic subunit of protein phosphatase 1 (PP1) (45), protein phosphatase 2A (PP2A) through its regulatory B subunit PR130 (45), casein kinase 1 delta and epsilon (CK1δ/ε) (52, 53), PKC isozymes (PKCβ, PKCδ, PKCε, PKCθ) (21, 54), calmodulin (55), the γ-tubulin ring complex (γ-TuRC) comprising of γ-tubulin, tubulin gamma complex associated proteins 2, 3, 4, 5 and 6 (TUBGCP2, TUBGCP3, TUBGCP4, TUBGCP5, and TUBGCP6) (55, 56), dynein/dynactin (57), Cdc42-interacting protein 4 (CIP4) (58), Ran (59), phosphodiesterase 4D (PDE4D) (45, 60), cyclin E/cyclin-dependent kinase (Cdk) 2 (61) and Golgin A2/GM130 (62) in various cell types (Figure 2). In cultured epithelial cells, CG-NAP forms a pericentrosomal complex with the EB1-binding-myomegalin protein complex and recruits calmodulin regulated spectrin-associated protein family member 2 and 3 (CAMSAP2 and CAMSAP3) and microtubule-associated protein RP/EB family member 1 (MAPRE1) to the Golgi (55, 63–66). Studies using various cell-types, including human T lymphocytes, further elucidate a role of CG-NAP in microtubule nucleation (20, 56, 67). These binding interactions with multiple proteins suggest a dynamic complexity in the active functions of CG-NAP.

In the context of T lymphocyte functions, crucial roles for CG-NAP have been demonstrated as (i) a component of the LFA-1-induced signaling complex, (ii) a mediator of T cell/APC immune synapse (IS) formation, (iii) an organizer of centrosomal re-localization, and *iv*) a facilitator of cytoskeletal rearrangement and motility (20–22). The LFA-1 β2 integrin plays multiple roles in the functioning of T lymphocytes, including migration to sites of inflammation/infection, proper functioning of the IS and functional programming for effector differentiation. In consequence, LFA-1-induced signals are critical in the pathogenesis of inflammatory diseases, such as psoriasis (68), and infectious diseases, including human immunodeficiency viruses (HIV) (69–71), Hepatitis C (72, 73) and John Cunningham (JC) virus (74). An active involvement of CG-NAP in mediating LFA-1 signaling suggests its potential implications for the above and other T cell-dependent diseases.

In prior studies, we have demonstrated that CG-NAP is an important component of the LFA-1 signaling complex for T lymphocyte migration (20, 21). These studies established that CG-NAP is expressed in T lymphocytes at the centrosome at rest and distributed both at the centrosome and along the trailing microtubules during migration (20, 21). CG-NAP co-immunoprecipitates with LFA-1 in activated migrating T cells (21). PKC isoforms, including PKCβ and PKCδ, also interact with CG-NAP in motile T cells (21). Hence, we concluded that the migratory signals in T lymphocytes induce the assembly of a multi-molecular protein complex involving CG-NAP, which serves as one of the docking platforms for PKCβ and PKCδ isoforms (21). PKCβ regulates LFA-1-mediated locomotion of activated T cells (75); whereas, PKCδ plays a critical role in TCR-induced negative regulation of IL-2 cytokine production and T cell proliferation (76).

Studies using cultured fibroblast cells demonstrated a direct association between CG-NAP and immature non- or hypo-phosphorylated PKCε at the Golgi and around the centrosome (54). Depending on environmental cues and upon phosphorylation, PKCε dissociates from the CG-NAP complex as a “mature” enzyme, which actively responds to second messenger signals (54). In human T cells, this PKC isoform regulates a diverse range of biological functions. In particular, PKCε modulates the TCR-associated signaling complex for T cell activation and cytokine secretion (77, 78), proliferation (79), sensitivity to TGFβ (79), development (80), gene expression (80), and survival (81, 82). PKCε directly activates the NFκB/NFAT/AP1 pathway in T cells leading to the up-regulation of IL-2 receptor expression and an increase in IL-2 production (83, 84). The LFA-1 signal for T cell migration activates PKCε, which phosphorylates the Rab GTPase Rab5a on Thr7, triggering a molecular cascade leading to the activation of the Rac1 protein and actin cytoskeletal rearrangements in motile T cells (85). Further studies are required to determine whether CG-NAP plays a role in the dynamic coordination of PKCε activities in human T cells.

### CG-NAP/Kinase Interactions in T Cells at the Immune Synapse (IS)

Upon recognition of specific antigen on APCs and TCR engagement, a T cell undergoes a series of structural and molecular changes to form a flattened contact site, termed the “IS” (86). Within few seconds of T cell/APC contact, TCR signaling is triggered *via* an array of phosphorylation and de-phosphorylation cascades of membrane-proximal and -distal signaling elements. Within few minutes, the T lymphocyte rapidly reorients its cellular content to the intercellular contact zone. In particular, the stimulated T cell repositions its centromere from the uropod to the synapse at the contact site and dynamically orients cytoskeletal systems that allow asymmetric segregation of signaling and adhesive proteins toward the APC contact (87). This centrosomal polarization is important for the directional movement of recycling TCRs to the IS (88) and the positioning of the T cell secretory vesicles toward the APC (89). These molecular processes facilitate the polarized secretion of cytokines and cytolytic factors toward the bound target cell for effector immune responses (e.g., cell-mediated cytotoxicity and target cell destruction) (90), while preventing undesired bystander effects on neighboring cells. A single T lymphocyte is thus able to eliminate multiple target cells consecutively by integrin-mediated adhesion, rapid rearrangement of contacts and simultaneous formation of stimulatory and lytic synapses with defined central and peripheral signaling platforms. Moreover, the IS facilitates cell-to-cell communication between the T cell and the APC through exosomes and microvesicles (91, 92). After several hours of contact, T cell undergoes functional activation (93), and eventually differentiates to effector or memory T cells.

In the context of IS formation, CG-NAP coordinates dynamic interactions between protein kinases and their substrates at the centrosome in T cells. It colocalizes with a range of signaling molecules with implications for both the central supramolecular activation cluster (c-SMAC), which includes the TCR/CD3 complex and various costimulatory receptors, and the peripheral supramolecular activation cluster (p-SMAC) that incorporates LFA-1 (22). Functional consequences of CG-NAP loss in T cells during the IS formation, either by overexpression of a dominant-negative form or siRNA-mediated knockdown, include (i) impaired conformational activation and positioning of LFA-1 at the IS, (ii) defective segregation of LFA-1 at the p-SMAC ring, (iii) impaired LFA-1-associated signaling, (iv) reduced expression of the TCR CD3ϵ chain with decreased activation and clustering of TCR at the IS, (v) reduced phosphorylation of CD3ζ (Y83) in the TCR/CD3 complex, (vi) impaired recruitment of PKCθ to the IS, (vii) diminished phosphorylation of the phospholipase C gamma 1 (PLC-γ1), (viii) reduced activation of intracellular adaptor proteins, including the linker for activation of T cells (LAT) and Vav1, (ix) reduced phosphorylation of ERK1/2, (x) delocalization of the centrosome, (xi) defects in the translocation of microtubule organizing center (MTOC) toward the IS, and (xii) diminished production of IL-2 (22). The PKCθ isoform, PLC-γ1, ERK1/2, Vav1, and LAT play critical roles in TCR signaling. For example, activation of the TCR triggers PKCθ-mediated phosphorylation of the Rap guanine nucleotide exchange factor 2 (RAPGEF2) at Ser960, which regulates the adhesiveness of LFA-1 to its ligand ICAM-1 *via* Rap1 (94). Essential roles of PKCθ in regulating TCR-induced NFκB activation in mature thymocytes, inducible gene expression program in T cells, up-regulation and clustering of the LFA-1 on the T cell surface, adhesion capacity of T cells, effector T cell functions and protection from T cell-mediated autoimmune reactions have been documented (80, 95–97). An impaired PLC-γ1 activation in CG-NAP depleted T cells would impair diacylglycerol production, which is important for dynein function and MTOC translocation (22). TCR-induced phosphorylation of both LAT and Vav1 is critical for the functioning of the c-SMAC complex (22).

In the context of cytoskeletal reorganization at the IS, CG-NAP facilitates microtubule nucleation at the centrosome and non-centrosomal regions in human T cells (20). It coordinates PKA-mediated phosphorylation of dynein in motile T cells (20), which is crucial for centrosome repositioning at the IS (87, 98). Following APC/T cell contact, CG-NAP interacts with the kinase CK1δ that phosphorylates the microtubule plus-end binding protein EB1, which increases microtubule growth speeds (99) and has consequences for the IS. For example, T cell cytoskeletal remodeling elicits the APC to mobilize its intercellular adhesive molecules (ICAM-1 and−3) and subsequently the MHC-II molecules at the IS (100). Moreover, CG-NAP loss in human T cells impairs actin polymerization (22), which is crucial for the stabilization of APC/T cell contact at the IS (101).

CG-NAP mediates the activation of Aurora A protein kinase in human T cells (102), which is crucial for regulating signaling downstream of the TCR, such as activation of the Lck kinase and opening of the Ca^2+^ release-activated channels (CRAC)—both key signals involved in antigen-dependent T cell activation and in IS formation. Interestingly both knockdown and over-expression of CG-NAP significantly inhibit IL-2 secretion (22), suggesting multiple overlapping effects.

Thus, T cell CG-NAP contributes to the formation and maintenance of IS by serving as an intracellular scaffold for kinases and facilitating the organization and activation of receptor molecules.

### CG-NAP/Kinase Interactions in T Cell Activation and Proliferation

The processes of T cell activation, proliferation and effector functions require several independent but coordinated molecular events initiated by TCR engagement (103). According to the clonal selection theory of adaptive immunity, the activation of a single lymphocyte clone provides sufficient function for an immediate immune response (i.e., proliferation of effector cells), as well as the regenerative capacity to maintain the selected lineage (i.e., development and differentiation of memory cells). In this context, CG-NAP is potentially involved in T cell proliferation and clonal expansion. While a direct role of CG-NAP in cell proliferation and cell cycle regulation has been identified in other cell types (51, 61, 104), further studies are required to dissect this role in T lymphocytes. Nonetheless some potential interactions may be inferred. For example, CG-NAP-depleted Chinese hamster ovary (CHO) cells and HeLa cells over-expressing C-terminus CG-NAP are arrested at the G1 stage of cell-cycle followed by the induction of apoptosis in these cells (51, 61, 104). It has been shown in CHO cells that CG-NAP, by anchoring cyclin E/Cdk2 to the centrosome, drives centrosomal amplification and cell cycle progression (61). Further studies are required to determine whether CG-NAP-cyclin/Cdk complexes are involved in T cell proliferation.

At the centrosome, CG-NAP interacts with the centrosomal protein 72 kDa (Cep72) (105) and recruits the Kizuna (Kiz) protein, which is phosphorylated by the polo-like kinase 1 (Plk1) (106). The phosphorylation of Kiz enhances its association with the CG-NAP interacting protein, pericentrin (106). This association galvanizes the pericentriolar material, facilitates microtubule nucleation on the centrosome and allows for tubulin polymerization at the plus-end of the microtubules (107). CG-NAP associates with the dynein/dynactin motor complex and together with kendrin/pericentrin, anchors γ-TuRC at the centrosome through binding to its TUBGCP2 and/or TUBGCP3 subunits at the amino terminal regions (55). It provides new nucleation sites for *de novo* microtubule polymerization (55, 67, 108), which is important for cell cycle progression and proliferation of T lymphocytes. While many components of these interactions have been identified in T cells, further studies are required to determine the precise mechanisms whereby CG-NAP can regulate proliferation in human T cells.

CG-NAP also plays a crucial role in the regulation of endosomal trafficking of the TCR and is required for the effective re-stimulation of T cells (109). CG-NAP-dependent signaling and endosomal trafficking are important for the retention of T cells at sites of inflammation in mice (109). However, the viability of CG-NAP-knockout mice and the normal T cell counts in mice with conditional deletion of CG-NAP (109) suggest that CG-NAP may largely be dispensable or redundant for the maintenance of the resting T cell complement in the mouse. Loss of CG-NAP function in T cells would thus impair their sustained activation and have immunological consequences (109).

It is now clear that repeated and transient contacts of effector T cells with APCs are needed to functionally activate T lymphocytes in tissues. This suggests that signal integration between successive contacts is necessary to achieve activation. In contrast, the interactions between naïve T cells and DCs in the lymph node are relatively less dynamic and, typically, such interactions last for several hours (110, 111). It has also been speculated that the mechanisms of T cell activation at inflammation sites may vary from the primary activation of naïve T cells in lymph nodes (109). These tissue-specific differences in T cell activation may explain why depletion of CG-NAP does not significantly affect baseline T cell presence and differentiation in lymphoid tissues but may significantly impact T cell re-activation under suboptimal antigen-presenting conditions, such as re-activation in inflamed non-lymphoid tissue (109). Nevertheless, it has now been established that CG-NAP interacts with kinases (such as PKCβ, PKCδ, PKCε, PKCθ) (21, 54) and protein phosphatases (such as PP1, PP2A) (45) and transduces important signals *via* TCR (22) to regulate tissue-specific T cell activation and proliferation.

### CG-NAP/Kinase Interactions in T Cell Migration

The recruitment of T cells to the tissue sites of infection or inflammation is critical to an effective immune response. Stimulated T lymphocytes leave lymphatic tissue and search the periphery for infected or transformed cells. When a T lymphocyte encounters an APC or a transformed cell, it mounts a specific immune response in a highly controlled manner. Blockade of the multi-step process of T cell motility can impair immune reactions, while uncontrolled migration can contribute to the development of autoimmunity.

In one pathway, T cell motility is dependent on the interactions between the T cell integrin LFA-1 and its ligand ICAM-1, which is expressed on endothelial surfaces during inflammation (112). LFA-1 engagement triggers a plethora of signaling cascades causing dynamic phosphorylation/de-phosphorylation of substrates by kinases and phosphatases and formation of macromolecular signaling complexes that culminate in cytoskeletal remodeling and T cell motility (112). CG-NAP is an integral component of these LFA-1-induced multi-molecular complexes and can serve to link the centrosome, microtubules and kinases, critical to the polarization and migration of T cells (5, 20, 21).

In human T lymphocytes, CG-NAP predominantly localizes in close proximity to the centrosome and the Golgi (20). This Golgi localization of CG-NAP is disrupted by microtubule depolarization (20). In HeLa-Kyoto cells, CG-NAP was found to recruit the microtubule cross-linking factor 1 (MTCL1), a molecule which crosslinks and stabilizes non-centrosomal microtubules to the Golgi membranes (113). Hence, we hypothesize a potential role of CG-NAP/MTCL1 interactions in T cell motility, a process which requires further investigation. Furthermore, as an AKAP, CG-NAP interacts with PKA and this complex consequently phosphorylates centrosomal proteins pericentrin and dynein in motile T cells (20). Dynein plays a crucial role in MTOC repositioning, cytoskeletal organization and the movement and processes of signaling complexes during T cell activation and motility (114, 115). In addition to PKA, several other kinases and phosphatases, including CK1, PKC, PP1 and PP2A, phosphorylate and dephosphorylate dynein in various cell types (116, 117) and all these enzymes are known to be anchored to the CG-NAP (45, 52, 53).

The functional significance of CG-NAP/kinase interactions for T cell motility is further underscored by the finding that the association between CG-NAP and LFA-1-induced signaling complex is greatly reduced when T cells are maintained at low temperature conditions (21). These data suggest a potential link between CG-NAP/kinase interactions and metabolic pathways in motile T cells. T cells overexpressing the C-terminal (aa 3699-3796) mutant form of CG-NAP fail to polarize and migrate (21). This CG-NAP C-terminus region contains the PACT (pericentrin-AKAP450 centrosomal targeting) domain, which binds additional proteins (50). For example, calmodulin binds to the C-terminus of CG-NAP in a calcium-independent manner (50). In addition, CG-NAP interacts with PKCβ and PKCδ (21), a process critical for signal transduction in motile T cells (75, 118). While *in vitro* knockdown of CG-NAP in human T cells significantly inhibited T cell migration and chemotaxis (20), no major impact of CG-NAP depletion on T cell motility was observed in T cell-specific CG-NAP knockout mice (109). The discrepancy between these findings could be attributed to the different model systems and experimental conditions.

It has been shown that the intracellular distribution of CG-NAP in LFA-1-stimulated motile T cells is different from that in cells stimulated to migrate through interactions with fibronectin (21). These data suggest that CG-NAP plays a unique role in β2 integrin-mediated T cell motility and may not have a similar role in adhesion and motility induced *via* different integrin families, such as, the β1 integrin. It has also been demonstrated that LFA-1-induced macromolecular signaling assemblies bring together molecules involved in intracellular transport and secretion. For example, LFA-1-induced formation of CG-NAP/kinase interactome containing PKCβ is crucial for T cell IL-2 production (21).

Microtubules are prominent elements of the cytoskeleton and dynamic cytoskeletal remodeling is essential for T cell motility. In addition to the cellular microtubule arrays emanating from the centrosome or the MTOC, secondary networks exist, in which microtubules are not anchored to the centrosome. While non-centrosomal microtubules are known to be present in differentiated cells (e.g., neurons, skeletal muscles, and epithelial cells), a recent report has demonstrated non-centrosomal microtubules emanating from CG-NAP in motile T cells (20). GapmeR-mediated knockdown of CG-NAP (119) disrupted both the centrosomal and non-centrosomal microtubule nucleation and inhibited post-translational tyrosination and acetylation of tubulin, illustrating the complexity of CG-NAP's role in coordinating microtubule dynamics and stability in migrating T cells (20).

## Prospects of Therapeutic Targeting of CG-NAP and Associated Challenges

Immune-mediated diseases caused by T cell dysfunction are an increasing cause of mortality worldwide. While available therapeutic agents target T cell trafficking and immune hyperactivity, such treatment modalities are often accompanied by significant side effects. For example, while blocking LFA-1/ICAM-1 interaction has been proven to be effective in treating immune diseases, such as psoriasis (120), such immunosuppression can trigger the activation of JC-1 virus in the central nervous system leading to the development of fatal progressive multifocal leukoencephalopathy (121, 122). In prior studies, we have demonstrated that pre-activation through the LFA-1 pathway also alters the T cell programme, such that these stimulated T cells become refractory to TGFβ-mediated suppression and exhibit increased IL-17 secretion (123). Further studies are required to delineate whether specific interactions between CG-NAP and its docking partners may mediate specific migratory or secretory signals impacting on immune effector mechanisms. Fine-tuning of these interactions can provide functional selectivity and may offer exciting therapeutic approaches for a wide array of immune-mediated diseases.

One such fine-tuning strategy could be to alter a specific CG-NAP/kinase interaction by targeting a single protein-protein interaction. This could be achieved by either developing inhibitors against a specific kinase interaction in the CG-NAP interactome or by targeting CG-NAP/kinase interacting domain by blocking peptides (124). Although more likely to serve as a research tool, blocking peptides may assist in designing and developing small molecules targeting CG-NAP/kinase interactions, representing an interesting area of research and drug discovery. Further structural modeling of CG-NAP/kinase interactions should also identify suitable targets for small molecule inhibitors.

Targeting CG-NAP in its entirety would be challenging mainly because (i) this adaptor protein is expressed as several alternatively spliced transcripts and (ii) the degree of complexity of CG-NAP's involvement in multiple aspects of T cell signaling makes it difficult to elucidate each of their individual roles. One plausible strategy to understand the role of CG-NAP in T cell functioning would be to selectively displace interacting kinases and their subtypes from the CG-NAP docking platform. This would require the development of isoform-specific disruptors and other molecular tools to dissect individual pathway and CG-NAP/kinase interactions with high specificity. Exciting tools are available to silence individual gene in T lymphocytes that can be used to study functional involvement of specific interaction between CG-NAP and an individual kinase. These include the use of antisense GapmeR (119), siRNAs, gene correction and CRISPR-Cas9 editing techniques, which can be employed to overcome immune-mediated pathologies.

## Conclusion

It is evident from the past two decades of research that CG-NAP regulates a plethora of biological processes by organizing supramolecular complexes and facilitating dynamic interactions between many different kinases and their substrates. In T cells, CG-NAP plays an important role in motility and participates in multiple interdependent pathways of T cell activation and effector functions. Thus, systematic studies are warranted to shed light on common or distinct binding partners and functions of CG-NAP and clarify to what extent CG-NAP/kinase interactions regulate T cell functions.

There is growing interest in developing protein-protein interaction disruptors, which would open new opportunities for therapeutic targeting of individual interactions between CG-NAP and specific kinases. A better understanding of CG-NAP/kinase interactions in T lymphocytes and their functional perturbations in immune response regulation is likely to lead to new frontiers in the treatment of T cell-mediated diseases.

## Author Contributions

NV and DK conceived the review idea. All authors contributed to the writing of this manuscript and approved the final manuscript version.

### Conflict of Interest

The authors declare that the research was conducted in the absence of any commercial or financial relationships that could be construed as a potential conflict of interest.
